# Effects of a *FCBP* gene polymorphism, location, and sex on Young’s modulus of the tenth primary feather in racing pigeons

**DOI:** 10.1038/s41598-022-05649-2

**Published:** 2022-02-02

**Authors:** Eberhard Haase, Andrzej Dybus, Aneta Konieczna, Alexander Kovalev, Stanislav Gorb

**Affiliations:** 1 Department of Functional Morphology and Biomechanics, Zoological Institute of the University of Kiel, Am Botanischen Garten 1–9, 24118 Kiel, Germany; 2 Department of Genetics, Faculty of Biotechnology and Animal Husbandry, West Pomeranian University of Technology in Szczecin, Aleja Piastów 45, 70-311 Szczecin, Poland

**Keywords:** Biological techniques, Biophysics, Genetics, Zoology, Materials science

## Abstract

Young's modulus (E) is a measure for stiffness of a material and a higher E means a higher stiffness. The respective polymorphism of the feather corneous beta-protein gene causes the replacement of glycine by cysteine. We looked for possible effects of the three *FCBP* genotypes on E in the 10th primaries of racing pigeons. However, we did not find a statistically significant difference of E between the genotypes, even within the sexes and/or within different locations under our test conditions. Our findings do not preclude the possibility that under other conditions (temperature, moisture) an influence of the glycine/cysteine polymorphism on E may exist. Compared to the more proximal locations of the rachis (base and middle) we observed lower values for E in the distal region (tip). The 10th primary constitutes the leading edge of the pigeon wing and this special function may require higher stiffness in the proximal parts of the shaft. We observed significantly higher values of E in females than in males, which result only from statistically significantly higher values in the middle region. The higher stiffness of female primaries may also contribute to the better results of hens compared to cocks in pigeon races.

## Introduction

To carry out their functions during flight, the shafts of avian primaries should be of low weight and tolerate a certain degree of bending without breaking. The primaries consist mainly of the protein ß-keratin^1,2^, which after new findings nowadays should be termed feather corneous beta-protein (FCBP)^3,4^. It is made up by ~ 100 amino acid residues and has a molecular weight near 10 kDa^2,5^. Like other corneous substances it exhibits a filament/matrix texture^2,5,6^. The framework of the filament has a helical structure with four repeating units per turn and a pitch length of 9.5 nm^5^. According to EM studies by Filshie and Rogers^7^ the diameter of the filaments (named microfibrils by them) is about 3 nm. They are embedded in the matrix material and the centre-to-centre separation is in the order of 3.5 nm. The two components, filament and matrix, are formed by a single protein^5^.

The rachis of primaries consists of a dense cortex and a foamy medulla^8–11^. These authors agree that the stability of the rachis is mainly based on the geometry of the cortex. The medulla contributes only 16.1% to the dorso-ventral stiffness and 7.8% to the lateral stiffness in pigeon primary shafts^11^, but it essentially reduces the weight of the rachis. For the calamus cortex, Earland et al.^12^ could show that in the interior two thirds the molecules are orientated parallel to the calamus axis whereas the exterior layer lies at right angles to the axis. Astbury and Bell^13^ observed longitudially directed polypeptide chains for the most part of the rachis and a thin outer layer running at right angles to this. Partial degradation of feathers by microorganisms^14,15^ could visualize fibres of 6–8 µm diameter which are arranged in the dorsal and ventral wall of the rachis cortex in three layers: a thick longitudinal layer adjacent to the medulla, a second layer surrounding the first one circumferentially which is covered by a thin third layer with longitudinally directed fibres. The lateral sides of the cortex, named epicortex, are formed by a cross-fibre architecture, thus enabling rigidity in torsion.

Most authors cited in the last paragraph concurrently state a neglecting role of the chemistry of the corneous material for the mechanical properties of the rachis. In chickens the frizzle locus, which causes curled feather rachis and barbs, is associated with a corneous region enriched with genes coding for FCBPs. Sequence analyses of the keratin gene cluster identified a 69 bp in frame deletion in a conserved region of KRT75, a keratin gene^16^. In domestic pigeons Dybus and Haase^17^ detected a polymorphism in the feather corneous beta-protein gene (feather beta-keratin gene) which causes the replacement of glycine by cysteine and vice versa. Cysteine residues can form disulfide bonds and thereby can play a crucial role for fibrous proteins. Thus, it seems possible that the polymorphism in the *FCBP* gene may affect the mechanical properties of primary feathers. In this paper we are going to study the influence of the *FCBP* gene polymorphism on mechanical characters by comparing rachides from racing pigeons carrying either Cys/Cys or Cys/Gly or Gly/Gly variants.

In many avian species of various orders striking differences in the plumage between the two sexes can be observed. This sexual dimorphism can concern size, shape, and colour of feathers. In some cases [e.g. mallards (*Anas platyrhynchos,* with curled feathers in the drake's tail) and chickens (*Gallus gallus,* sickle-shaped rectrices in the cock's tail)] gonadal hormones induce the dimorphism, but in other species different genetic mechanisms are involved^18–20^. To our knowledge no results concerning influences of sex on mechanical properties of primaries have been published. In our material, primaries from both female and male pigeons were investigated. Thus, in the present study, we aimed to detect possible differences affecting mechanical properties in the two sexes. This seems to be of special interest since female pigeons have been reported to show significantly better racing results than males^21^.

Cross sections of remige shafts look very different depending on the location of the cross sectiin^8,9^. Several authors observed local differences of mechanical properties in remiges of mute swan^11^, goose, swan^22^; pigeon, barn owl^23^ and contour feathers of chicken, turkey, ring-necked phaesant, herring gull^24^ with increasing values of Young’s modulus (E) from the base to the tip. No such differences were found in a primary of the ostrich^22^ and in the tail coverts (train) of the peacock^2^ hinting to the role of flying on local mechanical properties of feather rachides. Experimental recordings of in vivo strains on the shafts of various primaries and a secondary of flying pigeons indicate peak strain values in the 8th primary with a lower value in the 9th and a falling tendency among the more proximal primaries and the secondary^25^. The 10th primary forms the leading edge of the wing and it differs in morphological and mechanical properties from the 9th and more proximally primaries of pigeons^9^. In this paper we want to find out whether local differences in Young's modulus of the 10th primary correspond to the findings in other remiges or reflect in some way its special tasks.

## Results

Three-way ANOVA used to discriminate the effects of location (base, middle, tip), genotype (Cys/Cys, Cys/Gly, Gly/Gly), sex, and interactions between the mentioned factors on the value of Young's modulus showed no statistically significant difference between different *FCBP* genotypes even within different sexes and/or within different locations (p = 0.139).

**Figure 1:**
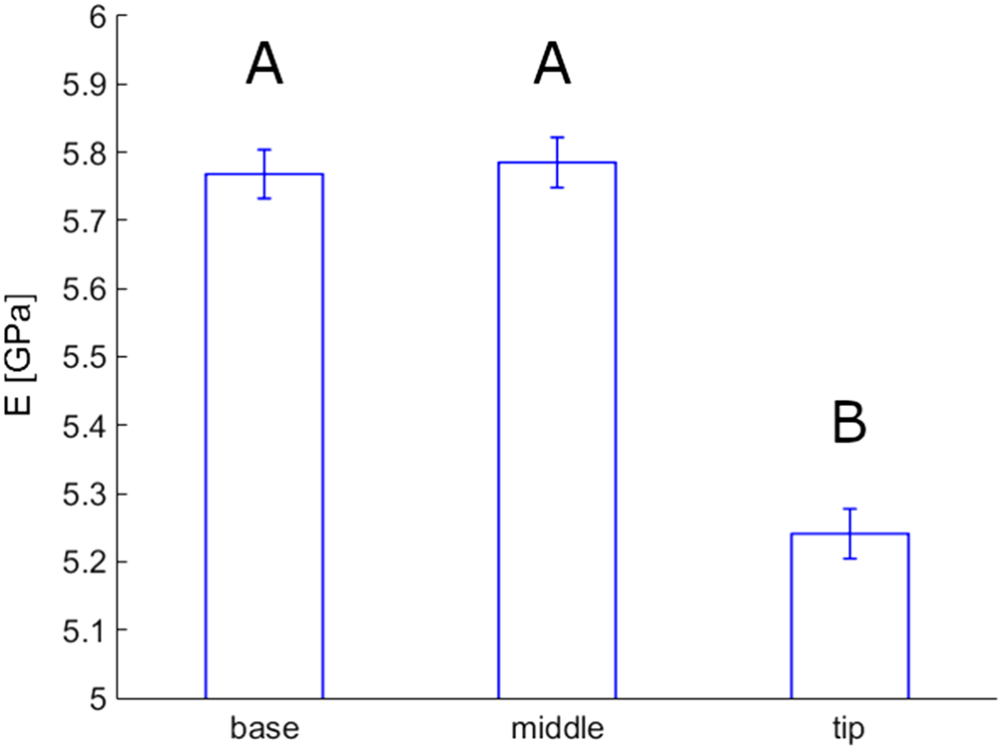
Effect of locations on Young's modulus values of feathers. Mean values (bars) and standard error (whiskers) are presented for base, middle, and tip regions of the feather shaft. 32 feathers from 32 animals were used for the comparison. The number of individual measurements in the above-mentioned regions was 572, 537, and 546, correspondingly.

Therefore, two-way ANOVA (regardless of genetic background) was performed (see Supplementary materials 1). It demonstrated a statististically significant effect of location in Young's modulus (p < 0.001) between tip (5.24 GPa) and base/middle regions (5.77/5.79 GPa) of the feathers (Fig. 1).

**Figure 2:**
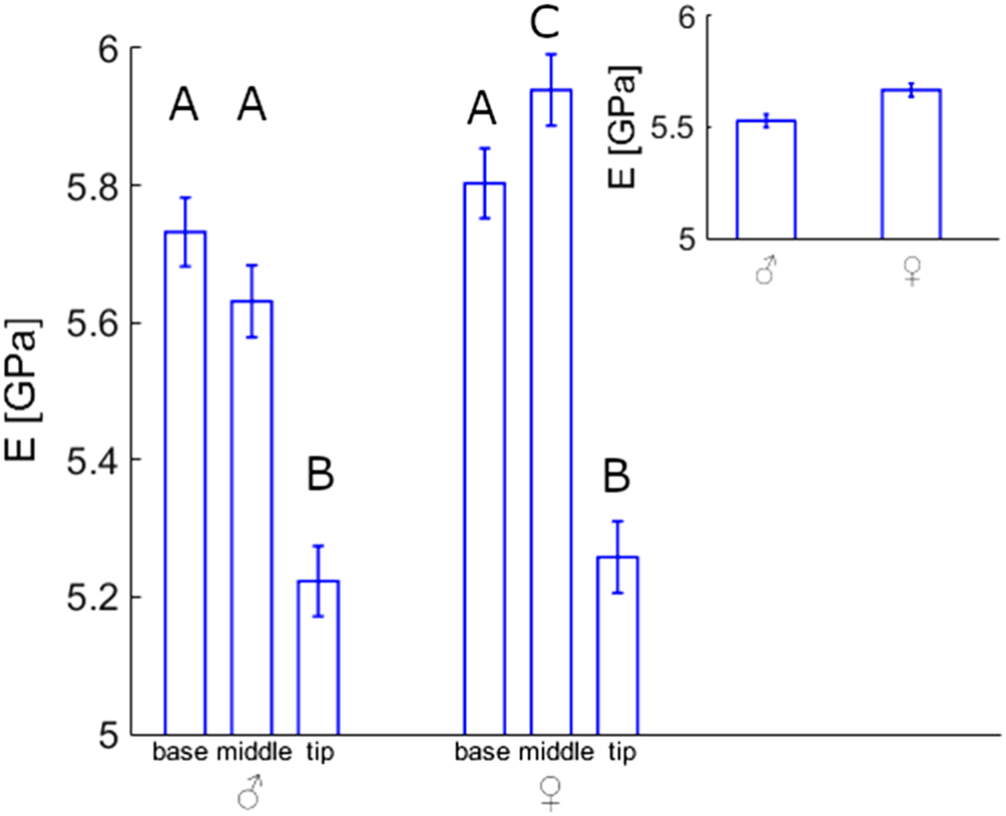
Effect of sex on Young's modulus values of feathers. 32 feathers from 32 animals were used for the comparison. The number of individual measurements in the base, middle, and tip regions was 291, 266, 278 for males and 281, 271, 268 for females, correspondingly. Comparison between males (835 measurements) and females (820 measurements) is shown in inset.

The same is true for the effects of sex. We found a statistically significant difference of E between females (5.67 GPa) and males (5.53 GPa). These different values result from the statistically significant difference only in the middle region of the feathers (females 5.94 GPa, males 5.63 GPa), whereas E either of the feather bases or of the feather tips show no such sexual difference (Fig. 2).

The Young's modulus in the rachis of the 10th primaries of our birds averaged between 5–6 GPa. This agrees well with the findings of Bachmann et al.^23^ on 5th primaries of pigeons and barn owls. These authors, using the nanoindentation technique as well, found no statistcally significant differences between the pooled mean values of the two species (pigeon: 5.96 GPa, barn owl: 6.54 GPa). Bonser and Purslow^11^ performed tensile tests on compact keratin cortex strips from the dorsal side of primaries in 8 avian species belonging to different orders, among them the rock pigeon, and found mean Young's modulus of 2.50 GPa in all species apart from the grey heron (E = 1.78 GPa). Bachmann et al.^23^ additionally applied bending tests on pieces of primary shafts of their pigeons and barn owls, the measured Young's modulus resembling those of the nanoindenter technique. The relative low values published for pigeons and other species by Bonser and Purslow^11^ might be due to the use of the tensile tests. In an earlier study, Purslow and Vincent^9^ estimated the stiffness of the cortex of pigeon rachis as 7.75–10 GPa by best fit to a bending model (see also Discussion in^23^).

## Discussion

### Corneous material composition and biomechanical properties

During the last 15 years new findings led to a new concept for the classification of corneous materials in vertebrates. The corneous structures in sauropsids like scales, claws, beaks, and feathers are essential formed by small proteins, formerly called beta-keratins (e.g.^1,2,5,6,15,17,26^), but nowadays called corneous beta-proteins (CBP). Genes coding for CBPs have evolved within the epidermal differentiation complex (EDC), a locus with no relationship with those of the IF-keratins (reviewed by Alibardi^3^ and Holthaus et al.^4^).

Among the various factors that might influence the mechanical properties of feather rachides (see “Introduction”) we focused in this paper on the chemical composition of FCBP. After studying the Young's modulus of primaries in eight avian species, Bonser and Purslow^11^ concluded that the flexural stiffness of the whole rachis in these species is principally controlled by their cross-sectional morphology rather than by material properties of the FCBP. This view was shared by Bachmann et al.^23^, who concluded that the flexural stiffness is predominantly influenced by the geometry of the feathers rather than by local material properties. The finding that within a single species, the domestic pigeon, and even within a single breed of it, the homing pigeon, a polymorphism in the feather corneous beta-protein gene was detected^17^ and offered the chance to further test a possible contribution of the chemical composition of FCBP on the mechanical properties in an otherwise very homogenous genetic background. This was even more tempting since the described polymorphism in the *FCBP* gene (*F-KER)* resulted in an interchange of cysteine and glycine. Cysteine is known for its ability to form disulfide bonds and its replacement by glycine would prevent the formation of these bridges and could thereby alter the stability of the protein molecule. Additionally, Proskura et al.^21^ observed a correlation between the racing performances of homing pigeons and their *FCBP* (*F-KER)* genotypes in races from different distances. From distances below 400 km the Gly/Cys birds returned faster than the other 2 genotypes, but this differences was statistically not significant. When released from distances of more than 500 km Cys/Cys pigeons homed with significantly higher speed than Gly/Gly birds.

However, in our measurements an influence of various *FCBP* genotypes on the Young's modulus of the rachis of the 10th primary could not be detected. Fraser and Parry^26^ have aligned the amino acid sequences of hard keratins (CBPs) in birds and reptiles. In birds, they found only minor variations in the chain lengths. Feather keratin molecules may be subdivided into three domains: a highly conserved central domain consisting out of 34 residues and the slightly more variable N-terminal and C-terminal domains. The central domain contains a high proportion of ß-favoring residues which are thought to be the framework of the filament. The framework of the filament is based on a pair of twisted ß-sheets related by a perpendicular diad. The ß-sheet consists of three internal strands and two shorter edge strands connected by four turns. Emu (*Dromaius novae-hollandiae*) feather keratin differs from that of other avian species. It has single insertions at the ends of the central domain and this may be related to the fact that it is a ratite (flightless) bird. If the amino acid sequence of the pigeons's polymorphic feather keratin^17^ is aligned to the system of Fraser and Parry^26^, the Cys/Gly polymorphic site turns out to be situated outside the central domain, but in the C-terminal domain (corresponding to position 172 in Fig. 4 in Fraser and Parry^26^) and thus it should not affect the framework of the filament.

The N-terminal and the C-terminal domains are thought to constitute the bulk of the matrix. The N-terminal domain has a high cysteine content and according to^26^ cysteine residues play a major role in rendering the assembled filament-matrix complex insoluble and rendering it resistant to attack by micro-organisms as well as influencing its mechanical properties. In the C-terminal domain of the avian feather keratins cysteine residues are sparse (Fig. 4, in^26^). In the great majority of avian corneus material molecules listed in this figure, including two pigeon sequences position 172 shows glycine, whereas in 344 domestic pigeons studied by Dybus and Haase^17^ the allele frequency of glycine (0.176) was much lower than frequency of cysteine (0.824). Whether this mutation results in an extra cystine linkage and/or influences the water content of the rachis is unknown. In their experiments, Taylor et al.^27^ found clear effects of hydration on the tensile and compressive properties of avian corneous material. In our measurements, temperature and humidity were kept constant and under these conditions an effect on the Young's modulus was not detected. This does not preclude a possible influence of the glycine/cysteine polymorphism on the mechanical properties of the pigeon rachis under varying humidity and temperature conditions.

### Local differences of Young’s modulus along the rachis

Young's modulus can vary along the length of the rachis. In a shaft of a mute swan, primary E increased approximately twofold from the base of the calamus to the tip of the rachis^11^. In 5th primaries of pigeons and barn owls, Bachmann et al.^23^ found significant differences of E between the proximal and distal feather parts in the two species, E of the proximal parts being significantly lower than E in the distal parts. In contour feathers taken from the pelvic tract of chicken, turkey, ring-necked pheasant, and herring gull E was found to be higher in distal than in proximal regions of the rachis both in bending and in tensile tests^24^. In tail feather coverts of peacock which function in sexual display but not in flight, Weiss and Kirchner^2^ observed no significant variation of Young's modulus with the position from proximal to distal. In wing feathers from the ostrich, a flightless bird, Cameron et al.^22^ found similar Young's modulus at 0, 50 and 75% of the total length of the rachis, whereas in the goose and in the swan, the values increased from the base to the tip. Different from the findings just cited in primaries and contour feathers of birds able to fly, we observed a slight decrease of E in the distal part of 10th pigeon primaries compared to the middle and the base region of the rachis. Purslow and Vincent^9^ detected differences in the morphology and in mechanical properties between the 10th and the 9th primary of pigeons. The 10th primary was equally stiff laterally as dorso-ventrally, whereas the 9th and other more proximally primaries were much less stiff laterally than dorso-ventrally. Cross-sections of the shafts show that the increase of lateral stiffness of the outermost primary is achieved by a greater width of the rachis compared to feathers proximal to it. These authors also point out that the outermost primary constitutes the leading edge of the wing. The other primaries lie behind the leading edge feather and are thus partly shielded by it. In adaptation to resist the high drag experienced by the 10th primary, it seems possible that an elevated Young's modulus in the basal and middle parts of this feather could be advantageous.

Corning and Biewener^25^ recorded in vivo strains on the shafts of the 9th, 8th, 6th, 5th, and 4th primary and the 2nd secondary of slowly flying pigeons using strain gauges attached to the dorsal side of the rachides approximately 2 cm distal to the calamus. Compressive strains during the downstroke exceeded tensile strains during the upstroke. The peak values were found in the downstroke of the 8th primary (− 0.0053). In the 9th it was − 0.0034 and in the 6th − 0.0036 with a falling tendency to the 2nd secondary (− 0.0021). These finding indicate that the different remiges experience different strains and consequently may vary in stiffness. Dorso-ventral deflexions of the shaft under static load applied at distances of 50% to 60% of the length resulted in higher values for the 10th than for the 9th primary (^9^, Fig. 6). Moreover, the 10th primary like the others is covered dorsally by the following feather but different from them has no protection from the ventral side. Also its vanes are very asymmetric with the distal one being extremely narrow. Regarding all these peculiarities of the outermost primary it will seem not so surprising that its local Young's modulus does not follow the pattern found in other remiges.

### Young’s modulus differs between the sexes

So far we found no sources describing biomechanical differences related to sexual feather dimorphisms. In several species of the order Columbiformes*,* both sexes look alike (monomorphic or monochromatic) and this also holds in the genus *Columba.* Looking more closely, the hens plumages sometimes seem to be duller and poorer in contrast compared to the males^28^, but often it is almost impossible to identify the sex of a rock pigeon, a feral or a domestic pigeon merely on the plumage. In spite of rather similar appearance of male and female pigeons, slight size differences have been described between the two sexes. Thus, Glutz von Blotzheim^29^ reports average body weights of 238.1 g for male and 231.5 g for female feral pigeons in Vienna and according to^30^, body mass and lengths of humerus, ulna and carpometacarpus differ in *C. livia* with males being bigger “. In our material the tenth primaries in males (185.3 mm) were longer than those of females (180.5 mm). It might be that these size differences are related to the sex differences in Purslow and Vincent^9^ studying primaries from five pigeons with body weights between 265 and 460 g reported that shape and size of the cortex, as measured by its second moment of area, have relations the body weight of the birds. On the other hand, Bonser and Purslow^11^ comparing primaries from seven avian species ranging in size from the common starling (60 g) to the mute swan (10 kg) found similar Young's modulus in these species.

Watching their behaviour is a better criterium to distinguish between sexes, but here, again, problems arise, since the behavioural differences between the sexes are rather quantitatively than qualitatively^31,32^. However, one behavioural trait, wing clapping, is mainly performed by the males. After copulation the male takes off loudly clapping his wings over his back for 3–5 wingbeats^30–32^. This makes a clapping sound^33^. This behaviour is part of the “display flight and it can also be observed in other situations as well, e.g. when a male notices another pigeon on the wing, especiallly if it is above him”, or “when he sees his mate or another pigeon in display flight nearby” or “when about to alight at or near his home after having been away foraging” and also “when flying in company with his mate” (^31^, p. 296). When he heavily courts a female, he may fly up for a few flaps clapping his wings or when the hen runs or flies away from him while she is courted he may follow her loudly clapping his wings (personal observation E.H. and A.D.). Slow motion videos from pigeons during take-off show that clapping during normal take-off results from primaries and secondaries that beat together over the birds back at the end of the upstroke^33,34^. During the upstroke the primaries are bent ventrally but at the end of the upstroke they become bent dorsally and meet the contralateral primaries over the bird's back. This bending may be facilitated by the lower stiffness (E) in the middle of the males shafts compared to females. Clapping related to courtship looks like an intensified version of off-take clapping and is primarily performed by the males.

In short (< 400 km) races as well as in long (< 500 km) races hens performed significantly better than cocks^21^. In conclusion our biomechanical findings are consistent with the idea that the higher stiffness of the females primaries contributes to the difference in the speed of homing.

## Methods and materials

### Study approval

This study was carried out in strict accordance with the recommendations of the National Ethics Committee on Animal Experimentation. The protocol was approved by Local Ethics Committee for Animal Testing of the West Pomeranian University of Technology in Szczecin (Protocol Number: 36/2012).

Homing pigeons from the lofts of A.D. and friends in Poland and E.H. in Kiel were used in this study. The birds had previously been genotyped for their *FCBP* alleles by A.D. using the method described in^17^ on DNA extracted from trunk feathers collected during the annual molt or from blood samples. Tenth (most distal) primaries of 32 adult (> 1.5 years old) birds (16 males, 16 females) were collected at the time of shedding in late fall and early winter. These feathers had grown about 12 months ago during the molting cycle of the previous year. 12 of these birds carried the *TT* or Cys/Cys *FCBP* genotype, 11 were heterozygous (*TG* or Cys/Gly) and the remaining 9 were homozygous (*GG* or Gly/Gly). In 27 birds the basic colour was black (wild type) showing different patterns: blue barred (+), checker (C), dark checker (C^T^), and uniformly black (S), in 5 pigeons the basic colour was red (ash red B^A^) with the patterns barred, check and dark check (nomenclature and genetic symbols after Sell^35^). In the first group the dominating pigment was eumelanin, whereas in the second group pheomelanin predominated^36^. Mean feather length was 185.3 mm in the males and 180.5 mm in the females.

Samples of the dorsal cortex of each rachis (2–3-mm long and 0.5–1.5 mm wide were cut from three regions: base (at the boundary between rachis and calamus), middle and tip (15% of feather length from the feather tip). These samples (2–3 mm long and 0.5–1.5 mm wide) were gently cut with a very sharp scalpel to avoid stress deformation of the material. For nanoindentation they were mounted to aluminum cylinders at room temperature with cyanoacrylate instant glue (ergo 5925 elastomer, Kisling AG, Wetzikon, Switzerland), which produces a very thin glue layer. The fixed samples were checked using a New View 4 k white light interferometer (Zygo, Middlefield, CT, USA) to determine the surface topography. Only areas with average surface roughness (R_a_ < 60 nm) were used for further nanoindentation measurements.

**Figure 3:**
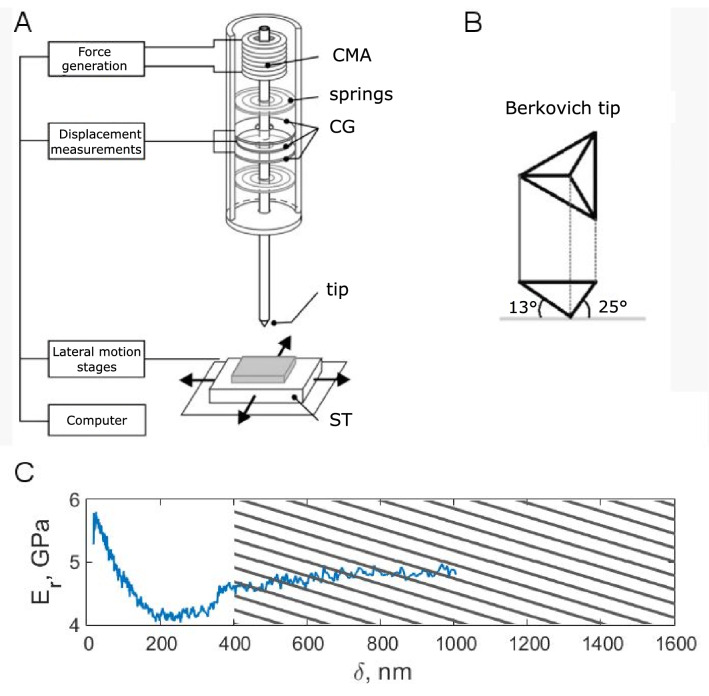
Nanoindentation experiments. (**A**) A scheme of the nanoindenter (adopted from Klein et al.^44^). (**B**) Berkovich tip imprints on a feather sample after nanoindentation. (**C**) CSM measurement curve of Young's modulus (E). The material parameters were averaged over the hatched area. CMA is a coil/magnet assembly, springs are supporting springs, CG is a capacitance gauge, tip is a Berkovich indentation tip, ST is a 2D moving/positioning stage with a sample tray.

Nanoindentation involves the application of a controlled load to the surface to induce local surface deformation using a sharp indenter tip with high elastic modulus, Fig. 3A. Smooth surface and well-defined geometry are required for the tip as well. The most used material for tips is diamond with an elastic modulus E = 1140 GPa. A three-sided sharply pointed Berkovich tip (the total included angle is 142.3°, a tip radius in our experiments was 150 nm, Fig. 3B) is more efficient over spherical, conical or pyramidal indenters, especially for a wide range of materials, including biomaterials.

With a 1 μN to 20 mN force range and 1 nm to 20 μm displacement range, nanoindentation bridges the gap between atomic force microscopy and macroscale mechanical testing. Because of its small probe size, nanoindentation can be used to measure local material properties in small, thin, and heterogeneous samples. Nanoindentation is also useful for measuring mechanical properties of microstructure within bulk samples, characterizing the properties of individual components within heterogeneous samples, mapping mechanical properties across a sample surface. This allows testing of samples unsuitable for other mechanical testing techniquesand makes the nanoindentation indispensable for mechanical testing of biomaterials. So, nanoindentation has been used to investigate the mechanical properties of radula teeth in gastropods^37^, skin by different snake species^38^, caries lesions in dentin^39^, etc.

Displacement in our nanoindenter (MTS nanoindenter II, MTS Systems Incorporation, Oak Ridge, USA) was monitored by capacitance gauge, while force actuation was provided through magnetic coils. A schematic of a nanoindenter system is shown in Fig. 3A. Such properties as Young’s modulus and hardness are calculated from the load–displacement curves using well-established equations based on elastic contact theory^40^. E can be calculated by the formula:

$$E=\frac{S}{2}\sqrt{\frac{\pi }{A}},$$

where *S* is the contact stiffness, and *A* is the contact area, which can be found from the dependance of contact area from the contact depth after tip shape calibration procedure using a standard material with well-known properties (e.g. fused quartz with E = 69.6 GPa)^41^. Finally, the Young’s modulus was averaged at penetratration depth excedding 400 nm to exclude the effect of the surface roughness on the measurements, Fig. 3C.

Dynamical Young’s modulus was determined using continous stiffness measurements method^42,43^ with nanoindenter controlling software Test Work 4 (MTS Systems Inc.).

### Statistical analysis

Two-and three-way ANOVA was performed with SigmaPlot 12.5^45^ (Systat Software, Inc. Erkrath, Germany). Data normality distribution and variance equality were checked before post-hoc analysis. Kolmogorov-Smirnow test was used for distribution normality check. Holm-Sidak method was used for post-hoc all pairwise multiple comparison procedure.

### Statement

Our study is reported in accordance with ARRIVE guidelines^46^.

## Supplementary Information

Supplementary Information.
