# Supplementary material for: Effects of a FCBP gene polymorphism, location, and sex on Young’s modulus of the tenth primary feather in racing pigeons
Source: Sci Rep. 2022 Feb 2;12:1785. doi: 10.1038/s41598-022-05649-2 (PMC8810990; doi:10.1038/s41598-022-05649-2)
Supplement: Supplementary file 1 — Supplementary Information. [file 41598_2022_5649_MOESM1_ESM.docx]

**Supplementary 1**

**Youngs’ modulus ANOVA**

Three Way Analysis of Variance

General Linear Model

Dependent Variable: E

Normality Test (Shapiro-Wilk) Passed (P = 0.061)

Equal Variance Test: Failed (P < 0.050)

Source of Variation DF SS MS F P

Location 2 99.133 49.567 68.097 <0.001

Sex 1 7.895 7.895 10.846 0.001

Genotype 2 2.875 1.437 1.975 0.139

Location x Sex 2 4.633 2.316 3.182 0.042

Location x Genotype 4 6.719 1.680 2.308 0.056

Sex x Genotype 2 0.586 0.293 0.402 0.669

Location x Sex x Genotype 4 4.825 1.206 1.657 0.157

Residual 1637 1191.542 0.728

Total 1654 1325.710 0.802

The main effects for Location cannot be properly interpreted since the size of the factor's effect depends upon the level of another factor.

The main effects for Sex cannot be properly interpreted since the size of the factor's effect depends upon the level of another factor.

The difference in the mean values among the different levels of Genotype are not great enough to exclude the possibility that the difference is just due to random sampling variability after allowing for the effects of differences in Location and Sex. There is not a statistically significant difference (P = 0.139).

The effect of different levels of Location depends on what level of Sex is present. There is a statistically significant interaction between Location and Sex. (P = 0.042)

The effect of different levels of Location does not depend on what level of Genotype is present. There is not a statistically significant interaction between Location and Genotype (P = 0.056).

The effect of different levels of Sex does not depend on what level of Genotype is present. There is not a statistically significant interaction between Sex and Genotype. (P = 0.669)

-------------------------------

Two Way Analysis of Variance

General Linear Model

Dependent Variable: E

Normality Test (Kolmogorov-Smirnov) Passed (P = 0.464)

Equal Variance Test: Passed (P = 0.202)

Source of Variation DF SS MS F P

Location 2 105.035 52.518 71.766 <0.001

Sex 1 7.859 7.859 10.739 0.001

Location x Sex 2 5.987 2.994 4.091 0.017

Residual 1649 1206.724 0.732

Total 1654 1325.710 0.802

Main effects cannot be properly interpreted if significant interaction is determined. This is because the size of a factor's effect depends upon the level of the other factor.

The effect of different levels of Location depends on what level of Sex is present. There is a statistically significant interaction between Location and Sex. (P = 0.017)

Power of performed test with alpha = 0.0500: for Location : 1.000

Power of performed test with alpha = 0.0500: for Sex : 0.896

Power of performed test with alpha = 0.0500: for Location x Sex : 0.591

Least square means for Location :

Group Mean SEM

Base 5.768 0.0358

Middle 5.785 0.0369

Tip 5.241 0.0366

Least square means for Sex :

Group Mean SEM

m 5.529 0.0296

f 5.667 0.0299

Least square means for Location x Sex :

Group Mean SEM

Base x m 5.732 0.0501

Base x f 5.803 0.0510

Middle x m 5.631 0.0525

Middle x f 5.939 0.0520

Tip x m 5.223 0.0513

Tip x f 5.258 0.0523

All Pairwise Multiple Comparison Procedures (Holm-Sidak method):

Overall significance level = 0.05

Comparisons for factor: Location

Comparison Diff of Means t P P<0.050

Middle vs. Tip 0.545 10.477 <0.001 Yes

Base vs. Tip 0.527 10.298 <0.001 Yes

Middle vs. Base 0.0176 0.342 0.732 No

Comparisons for factor: Sex

Comparison Diff of Means t P P<0.050

f vs. m 0.138 3.277 0.001 Yes

Comparisons for factor: Sex within Base

Comparison Diff of Means t P P<0.05

f vs. m 0.0709 0.992 0.322 No

Comparisons for factor: Sex within Middle

Comparison Diff of Means t P P<0.05

f vs. m 0.308 4.174 <0.001 Yes

Comparisons for factor: Sex within Tip

Comparison Diff of Means t P P<0.05

f vs. m 0.0346 0.472 0.637 No

Comparisons for factor: Location within m

Comparison Diff of Means t P P<0.05

Base vs. Tip 0.509 7.094 <0.001 Yes

Middle vs. Tip 0.408 5.560 <0.001 Yes

Base vs. Middle 0.101 1.392 0.164 No

Comparisons for factor: Location within f

Comparison Diff of Means t P P<0.05

Middle vs. Tip 0.682 9.248 <0.001 Yes

Base vs. Tip 0.545 7.466 <0.001 Yes

Middle vs. Base 0.136 1.870 0.062 No
